# Beyond Wait Times: Neighborhood Income Distribution Among Emergency Department Visits Assigned a Leave-Without-Being-Seen Disposition

**DOI:** 10.7759/cureus.113506

**Published:** 2026-07-28

**Authors:** Raeghan H O'Leary, Hannah Adams, Pam McDougall, Paul Atkinson, Kavish Chandra, Mark McGraw

**Affiliations:** 1 Medicine, Dalhousie University, Saint John, CAN; 2 Family and Emergency Medicine, Dalhousie University, Saint John, CAN; 3 Emergency Medicine, Horizon Health Network, Saint John, CAN; 4 Emergency Medicine, Dalhousie Medicine New Brunswick, Saint John, CAN; 5 Emergency Medicine, Dalhousie University, Saint John, CAN; 6 Emergency Medicine, Saint John Regional Hospital, Saint John, CAN

**Keywords:** emergency medicine barriers, healthcare disparity, leaving-without-being-seen, primary health care providers, socioeconomic factors

## Abstract

Objectives

Patients who leave emergency departments (EDs) without being seen may experience adverse outcomes and are recognized as a marker of strain in the health care system. This study described the characteristics of ED visits assigned a leave-without-being-seen (LWBS) disposition at a tertiary care ED and evaluated how demographic and visit-level characteristics varied across neighborhood income categories.

Methodology

This retrospective descriptive study included all ED visits assigned an LWBS disposition at Saint John Regional Hospital from January 1 to December 31, 2025. Socioeconomic status was assigned using Statistics Canada Postal Code Conversion File Plus (PCCF+) Version 8D and neighborhood income quintiles (QABTIPPE). Descriptive statistics were used to summarize patient characteristics. Categorical variables were compared using chi-square tests, and continuous variables were compared using Kruskal-Wallis tests.

Results

A total of 7,221 ED visits were assigned an LWBS disposition. Socioeconomic linkage was successful in 6,824 visits (94.5%). The median age was 36 years (interquartile range (IQR) 21-58), and 3,925 (54.4%) visits were listed as female in the administrative dataset. Among visits assigned an LWBS disposition with successful socioeconomic linkage, the number of visits was highest in the lowest neighborhood income category (2,225, 32.9%) and lowest in the highest neighborhood income category (846, 12.5%). Documented primary care provider (PCP) status varied significantly across neighborhood income categories. Visits from lower-income neighborhoods were more frequently missing a documented PCP in the administrative dataset than visits from higher-income neighborhoods (724, 32.8% vs. 125, 14.9%; χ²(4) = 158.6, *P* < 0.001). Median wait times to LWBS were similar across income categories, with no statistically significant difference (Kruskal-Wallis χ²(4) = 4.00, *P* = 0.406).

Conclusions

Among visits assigned an LWBS disposition, a larger number of visits originated from lower-income neighborhoods, and absence of a documented PCP was more frequent in lower-income categories. Median wait times were similar across neighborhood income categories. These findings describe socioeconomic patterns within the LWBS population and should not be interpreted as identifying factors that contribute to LWBS. Future studies incorporating comparator groups are needed to better characterize socioeconomic differences in LWBS visits.

## Introduction

Patients who leave emergency departments (EDs) without being seen are an important marker of health system performance, barriers to timely care, and ED crowding [1]. Recent literature describes that leave-without-being-seen (LWBS) should be considered within the broader context of ED crowding, where patient flow, prolonged wait times, and social determinants of health interact to influence access to emergency care [2]. Tropea et al. [1] found that for every $10,000 increase in household income, the odds of LWBS decreased. Individuals who experience LWBS experience higher short-term return rates and may face increased risk of adverse outcomes [3]. Social determinants of health, including socioeconomic status (SES), influence patterns of ED use, with lower-income populations relying more heavily on emergency services [4]. Early work by Stock et al. reported that 4.2% of patients in public hospitals left without being seen [5]. In comparison, recent New Brunswick Health Council data indicate a provincial LWBS rate of 11.5% for fiscal year 2023-2024 [6]. A large retrospective study from Ontario similarly demonstrated sustained increases in the frequency of LWBS visits from 2014 to 2023 and found higher post-LWBS hospitalization rates and short-term mortality during the post-COVID-19 period, despite lower total ED visit volumes [7].

Multiple studies have shown that LWBS is shaped by hospital crowding, prolonged wait times, difficulty in accessing primary care, socioeconomic disadvantage, and broader structural inequities within emergency care delivery [8-10]. Canadian evidence links socioeconomic deprivation with higher ED utilization and increased LWBS rates [11]. Individual-level characteristics associated with LWBS include presenting during off-hours, younger age, male sex, unstable employment, and visits related to mental health or substance use concerns [9,10]. Hsia et al. found that hospitals serving higher proportions of minimally insured patients had significantly higher LWBS rates, while communities with higher income had lower occurrences [8]. Sheraton et al. further identified poverty, insurance status, gender identity, and ED volume as key predictors of LWBS in a large U.S. dataset [10]. Although insurance status is less applicable in the Canadian context, these findings highlight the role of socioeconomic inequities in shaping patterns of LWBS.

Social marginalization may also contribute significantly to leaving the ED without being seen. Patients who leave before assessment are more likely to lack primary care attachment, stable housing, or reliable contact information, reflecting deeper systemic vulnerabilities [12]. In a mixed-methods study, McLane et al. found that First Nations patients in Alberta were nearly twice as likely to LWBS or leave against medical advice compared with non-First Nations patients, demonstrating the intersection of socioeconomic disadvantage and structural racism in emergency care [13]. Recent studies indicate that LWBS rates have increased substantially over the past decade. Fraser et al. found that long wait times were the most common reason for LWBS in two New Brunswick EDs, while concerns about personal health were the primary reason patients remained [14]. Tropea et al. similarly observed that LWBS was most strongly associated with time-based factors such as prolonged wait times, particularly in large urban EDs, rather than complex social variables [1]. In the United States, Janke et al. reported that the LWBS disposition nearly doubled between 2017 and 2021, while Hsia et al. identified LWBS as a key hospital performance indicator related to ED crowding and operational strain [8,15]. Improvements in ED flow and reductions in crowding may help mitigate the LWBS rate [5,8].

Despite the growing availability of Canadian health system data, few studies have examined the socioeconomic distribution of patients who LWBS using linked neighborhood-level socioeconomic measures. This study aimed to describe the demographic, visit-level, and neighborhood socioeconomic characteristics of ED patients assigned an LWBS disposition at the Saint John Regional Hospital ED.

## Materials and methods

Study design and time period

This retrospective descriptive study included all ED visits assigned an LWBS disposition from January 1, 2025, to December 31, 2025.

This study was approved by the Horizon Health Network Research Ethics Board (file number: 102431) with a waiver of informed consent for the use of de-identified administrative data.

Setting

The study was conducted at the Saint John Regional Hospital (SJRH), a tertiary care center in New Brunswick, Canada, with an annual ED census of 55,000 patients.

Population

All ED encounters assigned an LWBS disposition during the study period were included. LWBS was identified using the administrative ED disposition field, defined as an ED encounter in which the patient registered and was triaged, but left before initial assessment by a clinician. Encounters were excluded if they had missing or invalid postal codes, incomplete key demographic variables required for analysis, or duplicate records. Duplicate records were defined as registration errors resulting in multiple records for the same patient encounter.

Outcome measures

Variables extracted included age, sex, Canadian Triage and Acuity Scale (CTAS) category, date and time of arrival, mode of arrival (e.g., ambulance, walk-in, police), chief complaint category, disposition status, and residential postal code. Wait time to LWBS was calculated as the time from triage to departure. The recorded departure time reflected the time at which the patient was documented as having left the ED, which may not correspond to the actual time the patient departed. CTAS scores were grouped into high acuity (CTAS 1-2), moderate acuity (CTAS 3), and low acuity (CTAS 4-5) categories for analysis. Primary care provider (PCP) status was determined using the administrative PCP field within the ED dataset. Patients were classified as having a documented PCP when a provider was listed and as having no documented PCP when the field was empty. This variable was interpreted as documented PCP status rather than a definitive measure of primary care access. Arrival time was categorized into four periods: morning (06:00-11:59), afternoon (12:00-17:59), evening (18:00-23:59), and night (00:00-05:59).

SES was assigned using the Postal Code Conversion File Plus (PCCF+) Version 8D (Statistics Canada). Postal codes were mapped to dissemination areas using population-weighted probabilistic linkage. Neighborhood income categories were derived using quintiles (QABTIPPE), where Category 1 represents the lowest income areas and Category 5 the highest. This approach provides an area-level proxy for socioeconomic context and cannot be used directly to infer individual-level income.

Data analysis

Data were cleaned and analyzed using R statistical software (R Foundation for Statistical Computing, Vienna, Austria). Before socioeconomic linkage, postal codes were cleaned by converting entries to uppercase and removing spaces. SES was assigned using PCCF+ Version 8D, which linked postal codes to dissemination areas and assigned neighborhood income category using QABTIPPE. Income category was treated as a five-level categorical variable, with Category 1 representing the lowest-income neighborhoods and Category 5 the highest-income neighborhoods. Records with missing postal codes or income category values that were not able to be assigned were excluded from income-based analyses.

Descriptive statistics were used to evaluate patient and visit characteristics. Continuous variables were right-skewed and therefore summarized using medians and interquartile ranges (IQRs). Categorical variables (neighborhood income category, PCP status, arrival period, triage acuity, and presenting complaint) were summarized using frequencies and proportions to describe the distribution of observations across categories. Comparisons across neighborhood income categories were performed using chi-square tests for categorical variables, including PCP status, arrival period, and triage acuity. Wait time to LWBS was compared across income categories using the Kruskal-Wallis test. A *P*-value <0.05 was considered statistically significant.

Sample size

This was a retrospective study that included all eligible LWBS during the study period. The sample size was determined by the number of patient visits that met the study criteria.

## Results

A total of 7,221 ED visits with an LWBS disposition were included. Socioeconomic linkage using PCCF+ was successful in 6,824 visits (94.5%), and 397 (5.5%) were excluded due to missing or invalid postal codes. Median age was 36 years (IQR 21-58), and 3,925 (54.4%) visits were listed as female in the administrative dataset. Most visits occurred in the evening (18:00-23:59; 2,908, 40.3%), followed by the afternoon (12:00-17:59; 2,225, 30.8%), the morning (06:00-11:59; 1,163, 16.1%), and the night (00:00-05:59; 929, 12.9%). The majority of visits were triaged as low acuity (3,673, 51.2%) or moderate acuity (3,397, 47.3%). Triage acuity was similar across income categories, although a modest statistically significant difference was observed (χ²(8) = 16.6, *P* = 0.035). High-acuity presentation accounted for 109 (1.5%) of visits.

**Figure 1:**
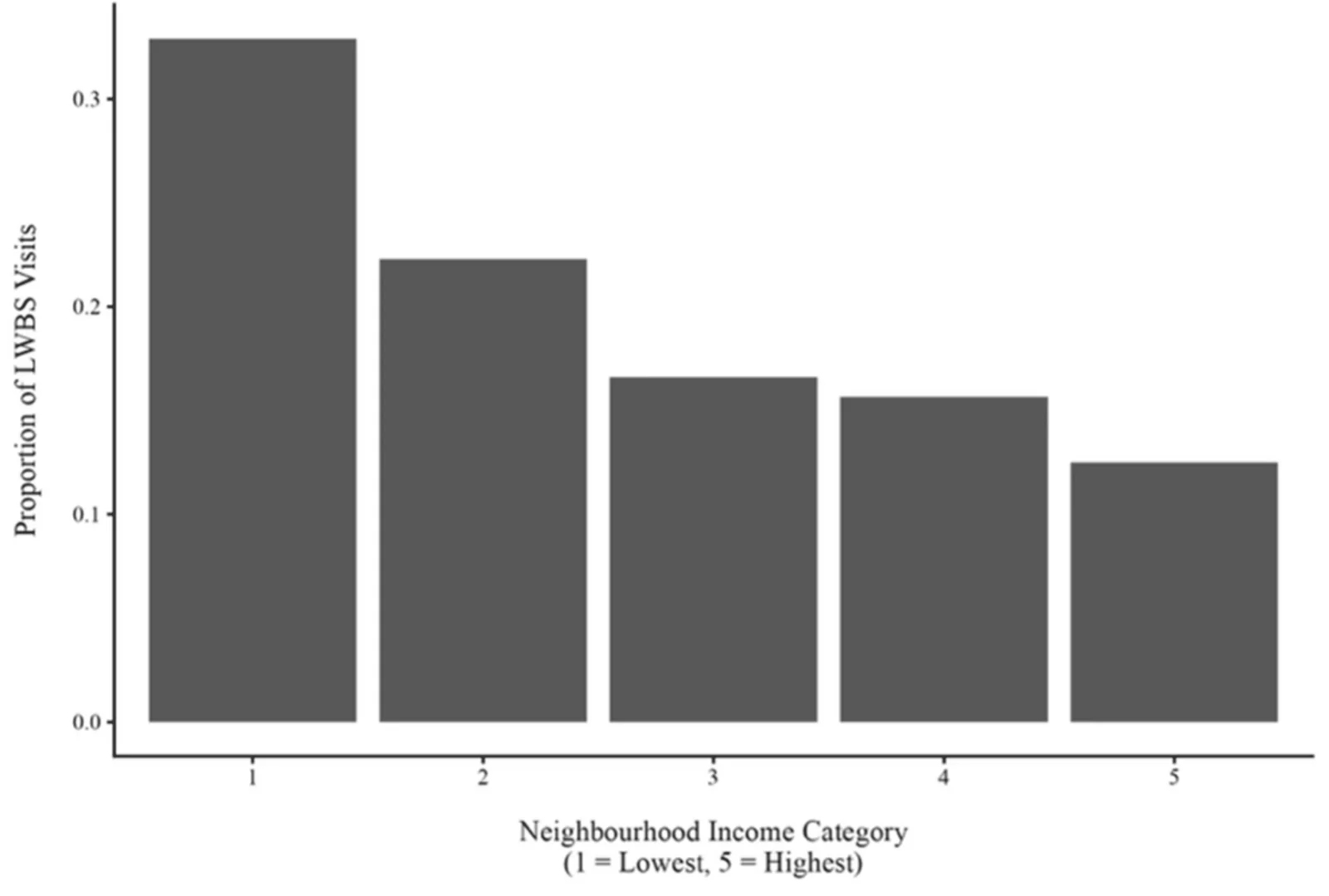
Distribution of leave-without-being-seen (LWBS) visits across neighborhood income categories. Data are presented as proportions (*n*/*N*). Corresponding visit counts and percentages were income category 1 (*n* = 2,225, 32.9%), category 2 (*n* = 1,507, 22.3%), category 3 (*n *= 1,123, 16.6%), category 4 (*n *= 1,060, 15.7%), and category 5 (*n *= 846, 12.5%).

**Figure 2:**
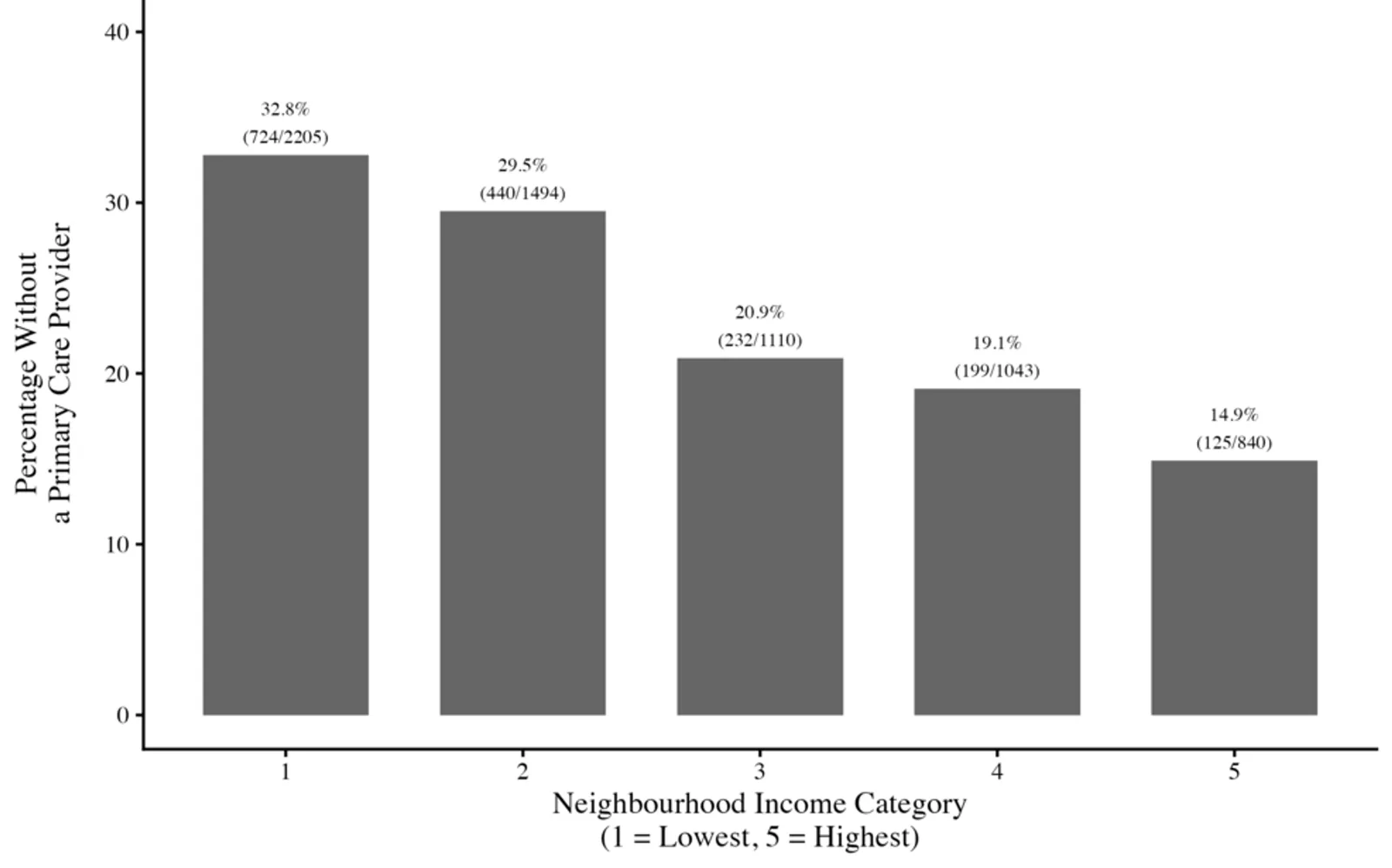
Percentage of leave-without-being-seen visits without a documented primary care provider across neighborhood income categories. Data are presented as percentages (%) with corresponding visit counts (*n*/*N*), where *n* is the number of visits without a documented primary care provider and *N* is the total number of visits in each income category. Chi-square test: χ²(4) = 158.6, *P* < 0.001.

Among patients assigned an LWBS disposition with successful socioeconomic linkage, the number of visits was highest from the lowest-income neighborhood category and decreased across higher-income categories (Figure 1). The lowest-income neighborhood category accounted for 2,225 visits compared with 846 visits in the highest-income category, representing more than a twofold difference in the distribution of LWBS visits. Documented primary care provider status varied significantly across income categories, with those from lower-income neighborhoods more frequently lacking a documented primary care practitioner than those in the highest-income group (724, 32.8% vs. 125, 14.9%) (chi-square test: χ²(4) = 158.6, *P* < 0.001) (Figure 2).

**Figure 3:**
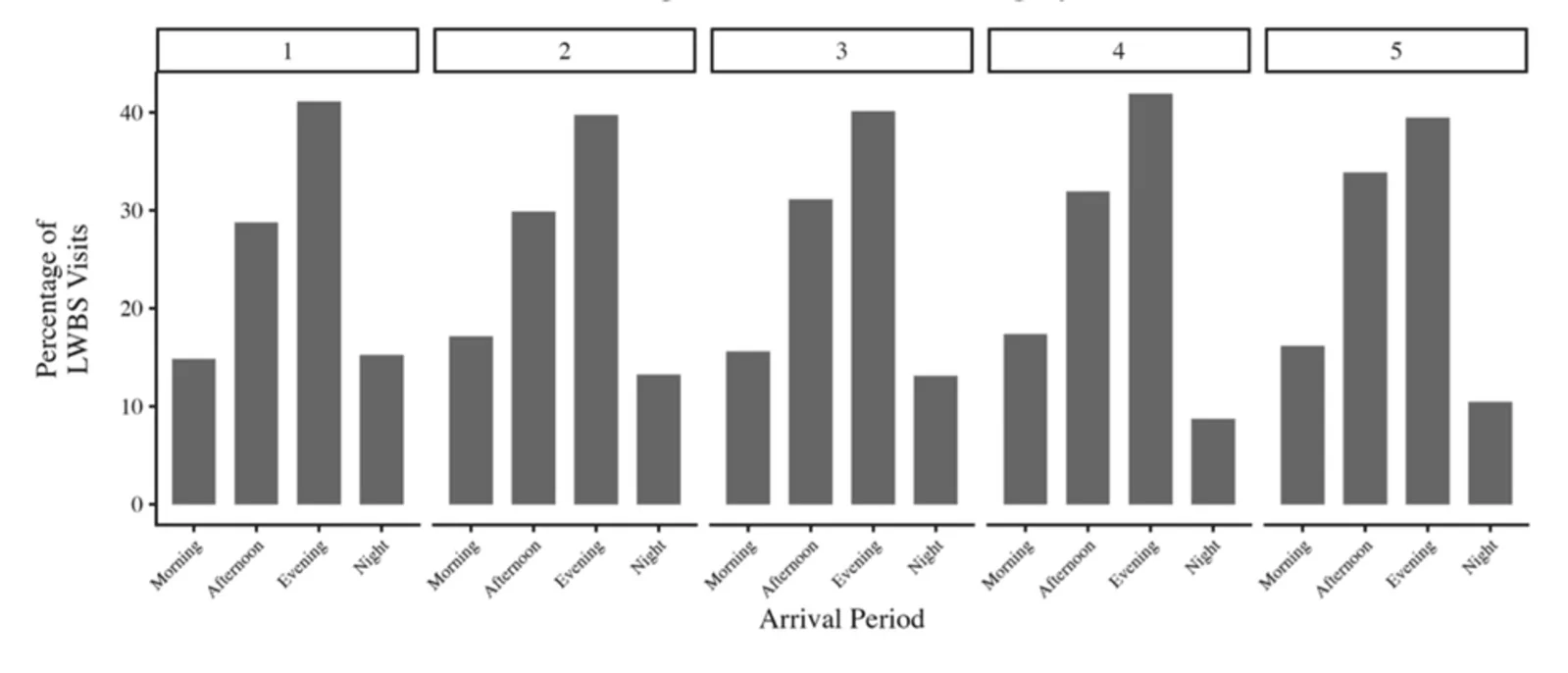
Distribution of arrival periods among leave-without-being-seen (LWBS) visits across neighborhood income categories. Data are presented as percentages (%). Chi-square test: χ²(12) = 39.9, *P* < 0.001.

**Figure 4:**
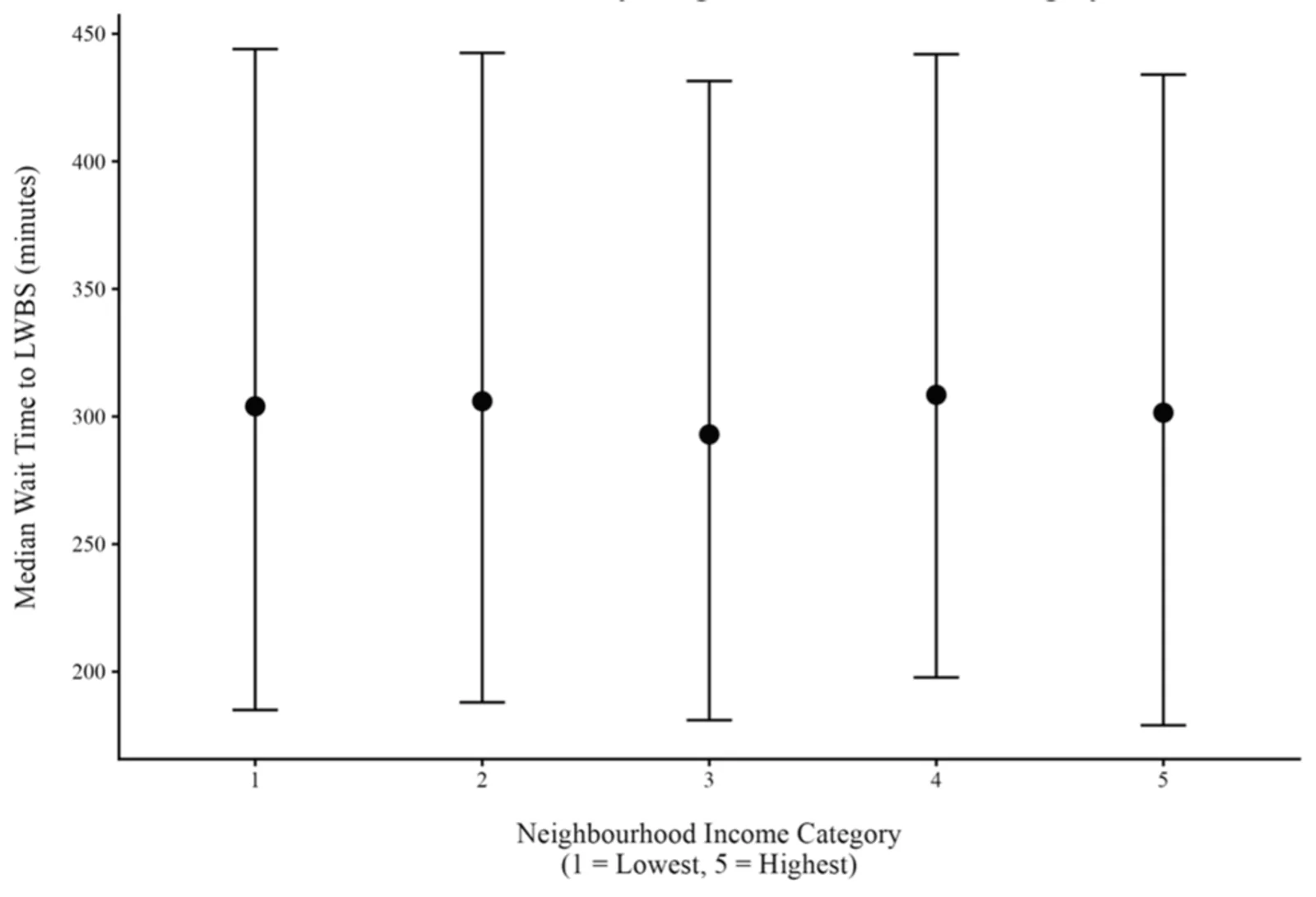
Median wait time to leave-without-being-seen by neighbourhood income category. Data are presented as median minutes with interquartile ranges. Kruskal-Wallis test: χ²(4)=4.00, p=0.406.

Evening presentation was most common across all income groups (Figure 3). The overall distribution of arrival periods differed significantly across neighborhood income categories (chi-square test: χ²(12) = 39.9, *P* < 0.001). Median wait times were similar across income categories and ranged from 293 to 308 minutes (Figure 4). There was no statistically significant difference in wait times between income groups (Kruskal-Wallis test: χ²(4) = 4.00, *P* = 0.406).

**Figure 5:**
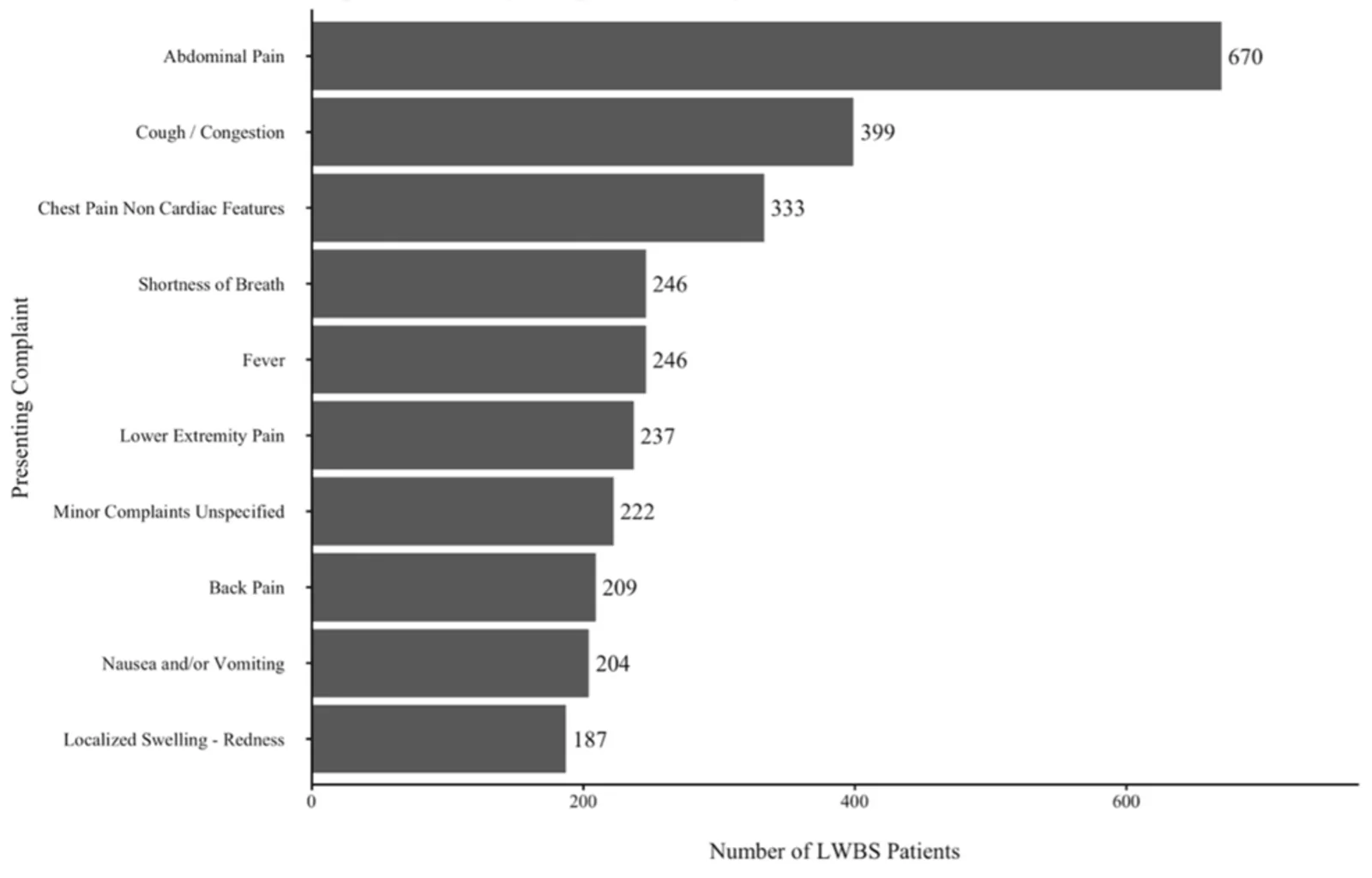
Most common presenting complaints among leave-without-being-seen (LWBS) patients. Data are presented as visit counts.

The three most common presenting complaints were consistent across all income categories: abdominal pain, respiratory complaints (cough/congestion), and chest pain with non-cardiac features (Table 1, Figure 5).

**Table 1:** Distribution of the 10 most common presenting complaints among leave-without-being-seen visits across neighborhood income categories. Data are presented as *n* (%).

Presenting complaint	Income category 1	Income category 2	Income category 3	Income category 4	Income category 5
Abdominal pain	219 (23.4%)	124 (20.6%)	116 (25.2%)	96 (22.7%)	73 (21.5%)
Cough/congestion	125 (13.4%)	73 (12.1%)	55 (11.9%)	62 (14.7%)	62 (18.3%)
Chest pain non-cardiac features	104 (11.1%)	60 (10%)	64 (13.9%)	53 (12.6%)	38 (11.2%)
Fever	58 (6.2%)	48 (8%)	42 (9.1%)	34 (8.1%)	39 (11.5%)
Shortness of breath	71 (7.6%)	57 (9.5%)	45 (9.8%)	31 (7.3%)	26 (7.7%)
Lower extremity pain	68 (7.3%)	61 (10.1%)	36 (7.8%)	33 (7.8%)	24 (7.1%)
Minor complaints unspecified	96 (10.3%)	43 (7.1%)	23 (5%)	24 (5.7%)	12 (3.5%)
Back pain	58 (6.2%)	55 (9.1%)	29 (6.3%)	39 (9.2%)	17 (5%)
Nausea and/or vomiting	67 (7.2%)	45 (7.5%)	32 (6.9%)	23 (5.5%)	28 (8.3%)
Localized swelling - redness	69 (7.4%)	36 (6%)	19 (4.1%)	27 (6.4%)	20 (5.9%)

## Discussion

Interpretation of findings

Our analysis of patients assigned an LWBS disposition indicates that a larger proportion originated from lower-income neighborhoods and the median time to departure was similar across all income categories. The higher proportion of patients without a documented PCP in lower-income neighborhoods is consistent with the possibility that primary care attachment may differ across socioeconomic groups, although administrative PCP documentation should not be interpreted as a definitive measure of primary care access. Variation in arrival patterns was also observed across neighborhood income categories, highlighting differences in the distribution of LWBS visits across socioeconomic groups.

Comparison with previous studies

ED utilization patterns have been previously linked to primary care attachment, with individuals lacking a PCP being more likely to use ED services for non-emergent complaints. In our study, the higher proportion of patients without a documented PCP in lower-income neighborhood categories is consistent with the possibility that primary care attachment may differ across socioeconomic groups.

Previous literature has identified LWBS as a marker of ED crowding and system strain and has linked it to prolonged wait times and adverse outcomes [1-3,8]. ED overcrowding is influenced by patient and systemic factors, such as barriers to primary care access, socioeconomic disparities, and delayed patient flow [2]. Most studies have focused on operational factors rather than socioeconomic determinants. Our findings add to existing literature by describing the neighborhood income distribution of LWBS using linked neighborhood-level socioeconomic data, highlighting the importance of considering equity-related factors when evaluating ED utilization.

Strengths and limitations

Strengths of this study are the PCCF+ socioeconomic linkage in a large ED cohort and the inclusion of multiple visit-level variables. Use of postal codes to assign neighborhood-level socioeconomic indicators is an established and accepted approach in Canadian health services research when individual socioeconomic data are unavailable [16]. The limitations of area-based socioeconomic measures, including potential individual-level misclassification, are well recognized and were considered in interpreting the results [17]. These methods allowed for a comprehensive evaluation of LWBS characteristics.

Our study has several limitations. First, this was a single-hospital study in a large urban center in New Brunswick, which may limit generalizability. While neighborhood-level characteristics have been used in previous studies to measure SES, they may not reflect individual-level income. The recorded LWBS time may not accurately reflect the actual time a patient left, as documentation may only have occurred after a staff member determined the patient was no longer present. Therefore, calculated wait times may overestimate the duration a patient was in the ED. Additionally, documented PCP status was based on the administrative data field and may not accurately reflect primary care attachment or appointment availability. Sex was also determined from provincial health cards and may not accurately reflect patient identities. The analysis was limited to univariable comparisons and did not account for potential confounding between demographic and visit-level characteristics. Therefore, the observed differences should not be interpreted as reflecting the independent effect of neighborhood income, as they may also be influenced by other characteristics, including arrival period and triage acuity.

 It should also be noted that the administrative dataset included ED encounters rather than unique individuals. Therefore, patients who left without being seen on multiple occasions may have contributed more than one observation to the study. Finally, the analysis was limited to patients assigned an LWBS disposition and did not include a comparator group of all ED patients. Therefore, the study describes the characteristics of patients who left without being seen but cannot identify predictors of leaving or estimate the likelihood of LWBS.

Research implications

To our knowledge, this is the first study to examine the neighborhood socioeconomic distribution and characteristics of patients assigned an LWBS disposition in a New Brunswick ED. Future studies including all ED patients and multivariable analyses are needed to determine whether neighborhood socioeconomic status is independently associated with leaving without being seen. A multi-center approach or a harmonized provincial data set would increase generalizability and better inform policy-level interventions to address inequities in healthcare access.

## Conclusions

Among patients assigned an LWBS disposition, a larger proportion originated from lower-income neighborhoods, and patients from these neighborhoods more frequently lacked a documented PCP. Wait times were similar across neighborhood income categories despite the observed socioeconomic patterns within LWBS visits.

These findings provide a descriptive characterization of patients assigned an LWBS disposition across neighborhood income categories. Future studies including all ED patients and multivariable analyses are needed to determine whether neighborhood socioeconomic status is independently associated with LWBS and better understand the mechanisms underlying these observed patterns.
